# Spatial-temporal distribution of neglected tropical diseases burdens in China from 2005 to 2020

**DOI:** 10.1186/s40249-024-01235-y

**Published:** 2024-09-04

**Authors:** Hanqi Ouyang, Ziyu Zhao, Ibrahima Socé Fall, Amadou Garba Djirmay, Okugbe Ebiotubo Ohore, Robert Bergquist, Guojing Yang

**Affiliations:** 1 NHC Key Laboratory of Tropical Disease Control, School of Tropical Medicine, the First Affiliated Hospital, Hainan Medical University, Haikou, 571199 Hainan China; 2 Global NTD Programme, WHO, Geneva, Switzerland; 3 Ingerod, Brastad, Sweden

**Keywords:** Neglected tropical diseases, Disability-adjusted life years, Spatiotemporal, People’s Republic of China

## Abstract

**Background:**

Out of the 21 neglected tropical diseases (NTDs) listed by the World Health Organization, 15 affect the People’s Republic of China. Despite significant achievements in controlling NTDs, comprehensive assessments of the disease burden based on actual case data and detailed information on spatial and temporal dynamics are still lacking. This study aims to assess the disease burden and spatial–temporal distribution of NTDs in China from 2005 to 2020, to provide a reference for the formulation of national health agendas in line with the global health agenda, and guide resource allocation.

**Methods:**

The number of cases and deaths of major NTDs in China from 2005 to 2020 were downloaded from the China Public Health Science Data Center (https://www.phsciencedata.cn/Share/index.jsp) of the Chinese Center for Disease Control and Prevention and relevant literatures. Simplified formulas for disability-adjusted life years (DALYs) helped estimate the years of life lost (YLLs), years lived with disability (YLDs), and total DALYs. Spatial autocorrelation analysis of the average NTDs burden data for the years 2005 to 2020 was evaluated using Moran's *I* statistic.

**Results:**

China's overall NTDs burden decreased significantly, from 245,444.53 DALYs in 2005 to 18,984.34 DALYs in 2020, marking a reduction of 92.27%. In 2005, the DALYs caused by schistosomiasis and rabies represent a substantial proportion of the total disease burden, accounting for 65.37% and 34.43% respectively. In 2015, Hunan and Sichuan provinces had the highest diversity of NTDs, with 9 and 8 number of different NTDs reported respectively. And the highest disease burden was observed in Sichuan (242,683.46 DALYs), Xizang Zizhiqu (178,318.99 DALYs) and Guangdong (154,228.31 DALYs). The “high-high” clustering areas of NTDs were mainly in China's central and southern regions, as identified by spatial autocorrelation analysis.

**Conclusions:**

China has made unremitting efforts in the prevention and control of NTDs, and the disease burden of major NTDs in China has decreased significantly. Using the One Health concept to guide disease prevention and control in the field to effectively save medical resources and achieve precise intervention.

**Graphical abstract:**

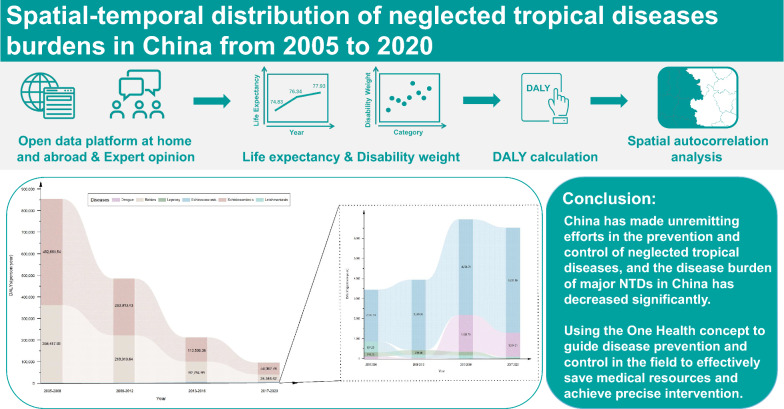

**Supplementary Information:**

The online version contains supplementary material available at 10.1186/s40249-024-01235-y.

## Background

Neglected tropical diseases (NTDs) are a group of diseases prevalent mainly in tropical and subtropical regions, widespread among populations in neglected, marginal, and impoverished communities. In 2010, the World Health Organization (WHO) published its first global NTDs report, “Accelerating Work to Overcome the Global Impact of Neglected Tropical Diseases—a Roadmap for Implementation”, in which 17 diseases were listed as NTDs [1]. In the new roadmap issued by the WHO in 2020, three diseases were added [2]. In 2023, WHO officially recognized noma as a new NTD [3]. The new roadmap also sets out overall global targets to be achieved by 2030: a 90% reduction in people requiring NTD interventions and a 75% reduction in the proportion of DALYs associated with NTDs [2].

Out of the 21 NTDs listed by the WHO, 15 affect the human health in the People’s Republic of China (Table 1). Since 1949, significant achievements have been made on the prevention and control of diseases in China. For instance, lymphatic filariasis was eliminated in 2007 [4]. By the end of 2014, the goal of eliminating blinding trachoma as proposed by the WHO was achieved [5]. Regarding *Schistosoma japonicum*, the transmission interruption was achieved by the end of 2023, with no acute reported human cases and no reported infection in *Oncomelania* snails for six consecutive years [6], so that the expected final elimination goal of schistosomiasis has been proposed by 2030 [2]. The prevalence rate of cystic and alveolar echinococcosis has decreased in recent years. However, a large population in China are still at risk of the infection, with significant regional and age distributions [7]. Leishmaniasis was primarily eliminated in China in 1958, but there have been local resurgences reported in northwestern and central China in recent years. Current incidence rates remain low [8]. Dengue has been reported in southern China, with most local cases associated with imported cases [9], which often develops into outbreaks [10]. In some regions, the prevalence and intensity level of food-borne trematodes (FBTs) infections, such as clonorchiasis, have expanded and increased both in space and time [11, 12].

**Table 1:** Presence and impact of and interventions against the neglected tropical diseases in China

Diseases (Category)	Pathogens	Number of infected persons (year)	Prevalence/weighted infection rate	Endemic areas	Transmission driver	Disability weight (DW)*	WHO goal	Chinese goal	Intervention launched
*Viruses*									
Dengue	Flavivirus Flaviviridae	778 (2020)	1.63/100,000^a^ (2019)	Southern PLADs	Common *Aedes* spp., and human movements	Post-dengue syndrome—0.219 Moderate dengue—0.051 Severe dengue—0.133 Dengue fever—0.81 (From the references)	No fatalities globally by 2030	Prevention & control in all communities by 2022	Mosquito breeding control Prevention of local transmission Promotion and education Vaccine/drug development
Rabies	Rhabdoviridae Lyssavirus	202 (2020)	0.037/100,000^a^ (2017)	All regions, mainly in the central and southern regions	Density of infected dogs	100% mortality—1.0	97% of countries having achieved zero human deaths from rabies by 2030	Absence of fatalities by 2035	Vaccination of humans & dogs Legislation & dog breeder control Promotion and education
*Bacteria*									
Leprosy	*Mycobacterium leprae*	200 (2020)	0.235/100,000^a^ (2015)	All regions, especially in the South	Exposure to lepromatous patients	Disfigurement level—1: 0.011 Disfigurement level 2—0.067	62% of countries with zero new autochthonous leprosy cases by 2030	Limiting transmission risk & cases by 2030	Early diagnosis & multidrug therapy Promotion and education Elimination of discrimination Multi-sectoral approach
Trachoma	*Chlamydia trachomatis*	—	—	All regions	Eye contact with contaminated water or hands	Blinding trachoma—0.581	Eliminated as a public health problem in 100% of all countries by 2030	Elimination was achieved in 2014	IEC, improved sanitation and water sources, and treated patients
*Fungus*									
Mycetoma, chromoblastomycosis, and other deep mycoses	Nocardia and Madurai mycetoma	—	—	—	Actinobacteriaceae bacterial or fungal infections caused by trauma	—	15 countries integrating mycetoma into national control programmes and surveillance systems by 2030	—	Personal hygiene and self-care of infected limbs; wear protective clothing to control sporotrichosis in pets to prevent transmission to humans
*Nematodes*									
Soil-transmitted helminths (STHs)	*Ancylostoma duodenale*, *Necator americanus*, *Ascaris* and* Trichuris*	Hookworm 16.97 million^b^; Ascaris 8.82 million^b^; Whipworm 6.6 million^b^ (2015)	Hookworm 2.62%^c^; Ascaris 1.36%^c^; Whipworm 1.02%^c^ (2015)	Southern and central parts of the country	Sanitation problem coupled with defecation outside latrines	Mild anaemia—0.004 Moderate anaemia—0.052 Severe anaemia—0.149 Heavy infestation—0.027 Mild abdominal signs—0.011 Severe wasting—0.128	Eliminated in 96% of all countries by 2030	Infection rates to decrease by more than 20% compared with 2015	Regular surveillance Control animal infection Drug-based de-worming Promotion and education
Lymphatic filariasis	*Wuchereria bancrofti*	—	—	—	—	Causes acute gonadal lymphangitis—0.051 Cause effusion—0.128 Cause lymphedema—0.109	81% of countries validated for elimination as a public health problem by 2030	Elimination was achieved in 2007	Regular surveillance
*Trematodes*									
Schistosomiasis	*Schistosoma japonicum*	29,522 cases of advanced schistosomiasis (2020)	—	Endemic in 12 provinces south of Yangtze River Basin	Unprotected water contacts in endemic areas	Acute schistosomiasis 0.13 Chronic schistosomiasis 0.192 Advanced 0.442 ± 0.162 For 0–14 years 0.098 For ≥ 15 years 0.198	Eliminated as a public health problem in all endemic countries by 2030	Expected final transmission elimination goal to be reached by 2030	Provision of safe water; Strengthen sanitation Improved diagnostics Continue snail control Promotion and education
Food-borne trematodes (FBTs)	*Clonorchis sinensis*	Clonorchiasis 5.98 million^b^ (2015)	Clonorchiasis 0.47%^c^ (2015)	All regions but mainly in the South and the Northeast	Consumption of raw or undercooked freshwater fish and shrimp	Over all for males 0.101 Over all for females 0.050 For 5–14 years—0.022 For 15–29 years—0.052 For 30–44 years—0.072 For 45–59 years—0.094 For > 60 years—0.118	12% of countries with intensified control in hyperendemic areas by 2030	Eliminated as a public health problem in all endemic countries by 2030	Prevention and treatment Provision of safe water Monitoring and protection Promotion and education
*Tapeworms*									
Taeniasis and cysticercosis	*Taenia solium* *T. saginata, T. asiatica*	Taeniasis 370,000^b^ (2015)	Taeniasis 0.06%^c^ (2015)	Northeast, Northwest and Southwest	Consumption of undercooked contaminated beef or pork	Central nervous system 0.471	27% of countries under enhanced control in endemic areas by 2030	National control and prevention plan criteria of 2019 achieved	Monitoring & early warning Food hygiene & quarantine Training of staff Change bad eating habits Improved pig management
Echinococcosis (alveolar and cystic)	*Echinococcus granulosus, E. multilocularis*	3327 (2020)	0.28%^d^	Endemic in 21 PLADs	Contact with livestock or consumption of contaminated vegetables or water	1. Average—0.302 2.Asymptomatic 0.257 3.Mild 0.280 4.Moderate 0.359 5.Severe 0.363	Increase number of countries with enhanced control in high-endemic areas from1 to 17	All endemic counties under control of key parasitic diseases by 2030	Screening and prevention Monitoring and recording Control of livestock slaughter Dog de-worming Sanitation and safe water Health education
*Other parasites*									
Scabies and other ectoparasitic diseases	Sarcoptes scabiei var hominis	—	—	Not reported in particular detail	Direct contact with infected people or animals, contaminated bed linen, clothing, etc	0.027	By 2030, all countries will include scabies management in their universal health coverage packages	—	Strengthen the prevention and control of pests, maggots and wild animals Improve the parasite monitoring system Pay attention to personal hygiene
*Protozoans*									
Leishmaniasis (visceral and cutaneous)	*Leishmania donovani, Leishmania infantum*	202 (2020)	—	Several PLADs in the Northwest and West	Sandfly	Moderate visceral 0.051 (0.032–0.074) Severe visceral 0.133 (0.088–0.19) Cutaneous 0.067 (0.044–0.096)	Visceral: 85% of all cases are detected and reported and 95% of reported cases are treated in 87 countries; Cutaneous: 85% of countries worldwide hsving eliminated kalazar by 2030	Reduce all parasitic diseases by 2020	Vector control & virus detection Vaccine development Screening of high-risk groups Encourage use of impregnated long-lasting mosquito nets Stop wildlife trade
*Toxins*									
Poisonous snake bites	Venom of snake	—	—	Southeast of China	Rural and remote areas	—	Reduce mortality and disability from snakebites by 50% by 2030 (WHO-Snakebite envenoming—A strategy for prevention and control)	Make snakebites a "global health priority" and halve the number of deaths and disabilities from snakebites over the next 12 years (WHO-Snakebite envenoming—A strategy for prevention)	Improve the monitoring and reporting system for snakebites Improve the professional level and treatment ability of medical staff
*Other microorganisms*									
Noma (cancrum oris)	Non-specific polymicrobial organisms	—	—	relatively rare	Extreme poverty, malnutrition, poor oral health, weakened immune systems	Not specified, but survivors often face severe functional and aesthetic impairments	Specific goals not available, but the WHO recognizes Noma as a neglected tropical disease, indicating a focus on control and eradication	—	Early detection, improving nutrition, maintaining basic hygiene, and antibiotic treatment to halt early-stage progression

*IEC* information, education, and communication strategies, *PLADs* provincial-level administrative divisions, *T.* Taenia, *E.* Echinococcus.

^*^The DW is assigned for diseases depending on the severity of the disability produced and presents disability weights that vary between 0 and 1, where the former represents good health and the latter is mortality or a state close to death

^a^Estimate the number of infected persons

^b^Estimated prevalence

^c^Weighted infection rate

^d^Prevalence. These annotations distinguish different types of epidemiological data presented in the table and appear in the "Number of infected persons" and "Prevalence/Weighted infection rate" columns in this table

Although China has made substantial progress in controlling NTDs, there is still a risk of zoonotic diseases and the emergence/re-emergence of a range of vector-borne infectious diseases. Even if schistosomiasis mansoni, schistosomiasis haematobium, African trypanosomiasis, dracunculiasis, American trypanosomiasis, onchocerciasis, Buruli ulcer and yaws have not occurred in China [13], the presence of certain vectors or intermediate snail hosts should be existed. For instance, *Triatoma rubrofasciata,* a vector of American trypanosomiasis, is widely distributed in southern China such as Guangdong and Hainan provinces [14]. A risk assessment considered China as moderate potential transmission areas of American trypanosomiasis [15]. *Biomphalaria straminea*, the intermediate snail host of *Schistosoma mansoni*, was found in Shenzhen City of southern China in the 1990s and has expanded widely in recent years [16].

The prevalence of NTDs in China has lately been the focus of intensive control programmes and multi-sectoral cooperative efforts. Its total burden has indeed declined significantly in the last decades. However, understanding the burden of NTDs in China was mainly based on global estimates and analyses. Although studies by Zeng et al. [17] and Yang et al. [18] provided valuable insights into the global and regional prevalence of NTDs, the detailed spatiotemporal analyses specific to provinces and regions within China are still missing. Furthermore, the latest NTD roadmap, which the WHO recently released, adds four new diseases. Given these major updates, conducting a comprehensive and updated assessment of the status of all NTDs within China is imperative.

This study aims to assess the disease burden and spatial–temporal distribution of NTDs in China from 2005 to 2020. With the up-to-date assessment of the burden of diseases, the study will provide an understanding of health challenges and play a key role in shaping national health agendas in line with the global health agenda, guiding resource allocation.

## Methods

### Data collection

The reported cases and deaths of dengue fever, rabies, leprosy, echinococcosis, and visceral leishmaniasis (kala-azar) from 2005 to 2020 was extracted from the Data-center of China Public Health Science of Chinese Center for Disease Control and Prevention (China CDC) (https://www.phsciencedata.cn/Share/). Data on schistosomiasis cases in China from 2005 to 2020 were obtained from the National Schistosomiasis Reports, available through the China National Knowledge Infrastructure (CNKI). Data for taeniasis/cysticercosis, clonorchiasis, and soil-transmitted helminths (STHs) were collected only for the year 2015 and were obtained from China's third national parasitic disease survey report [19]. The above data covers all Chinese province-level administrative divisions (PLADs) consisting of 31 Chinese provinces, autonomous regions, municipalities, Xinjiang Production and Construction Corp.

We referred to clinically relevant literatures to determine the disease course. Life expectancy and population data of each PLAD in China were obtained from the China Statistical Yearbook (https://www.stats.gov.cn/sj/ndsj/2021/indexch.htm). Disability weights (DW) for each disease were sourced from relevant literatures and Global Health Data Exchange (GHDx) (https://ghdx.healthdata.org/record/ihme-data/gbd-2019-disability-weights). We did not evaluate the DALYs of poisonous snake bites, noma (cancrum oris), scabies and other ectoparasitic diseases, as well as mycetoma, chromoblastomycosis and other deep mycoses due to lack of DW values and the complete number of cases. Trachoma and lymphatic filariasis have been eliminated in China.

### DALY calculation

This study uses a simplified DALY formula that does not consider age weighting and time discounting to estimate the disease burden from 2005 to 2020. Following the methods proposed by WHO [20], the original incidence or prevalence data for each disease are collected, processed, and analysed to calculate the years of life lost (YLLs) and years lived with disability (YLDs), and then summed to obtain the DALY measure according to the formula:

1 $$ {\text{DALY }} = {\text{ YLL }} + {\text{ YLD}};{\text{ YLL }} = {\text{ N }} \times {\text{ L}};\quad {\text{ YLD }} = {\text{ I }} \times {\text{ d }} \times {\text{ DW }} = {\text{ P }} \times {\text{ DW}}. $$

where I is the number of incident cases; d is the average duration of disease; DW is the disability weight; P is the number of prevalence cases; N represents the number of deaths; and L is standard life expectancy at age of death in years.

Dengue fever and rabies were calculated by I × d × DW. The rest NTDs were calculated by P × DW.

The changes in the disease burden for dengue, rabies, leprosy, echinococcosis, schistosomiasis, and visceral leishmaniasis from 2005 to 2020 were analysed and stratified into four timeframes: 2005–2008, 2009–2012, 2013–2016, and 2017–2020. For STHs, taeniasis/cysticercosis, and clonorchiasis, only nationwide epidemiological survey data for 2015 was available and retrieved; therefore, it was excluded from the comparative spatiotemporal analysis while calculating the overall and provincial disease burden for these diseases.

Similarly, DALYs data in 2015 were used for visualized analysis when comparing four diseases of canine origin (rabies, echinococcosis, leishmaniasis and clonorchiasis) and four diseases that could be treated with praziquantel (cysticercosis, schistosomiasis, echinococcosis, and clonorchiasis).

### Spatial autocorrelation analysis

Spatial autocorrelation analysis encompasses Global Indication of Spatial Autocorrelation (GISA) and Local Indication of Spatial Autocorrelation (LISA). The global Moran's *I* detects spatial clustering patterns across the study area. Subsequent local spatial autocorrelation analysis reflects the degree of correlation between a region and its neighbouring areas. Based on the *Z*-test (*P* ≤ 0.05), a LISA cluster map is constructed [21], thereby determining the spatial distribution of study units in "high–high", "high–low", "low–high", and "low–low" categories. The Moran scatter plot is utilized to examine local spatial instability.

### Statistical analysis

Data preparation was conducted using Excel 2021 (Microsoft, Redmond, American), while spatial data management and cartographic representation were performed using ArcGIS 10.8 software (Environmental Systems Research Institute, RedLands, American). Spatial autocorrelation analysis of the average NTDs burden data for the years 2005 to 2020 was carried out using GeoDa 1.2.0 (Center for Spatial Data Science, Chicago, American) software. Spatial autocorrelation was evaluated using Moran's *I* statistic. *P* values less than 0.05 were considered statistically significant. Additionally, the ranking changes of six diseases were visualized using the Origin 2022 (OriginLab, Northampton, American) software.

## Results

### The latest basic information on NTDs

Table 1 meticulously outlines the current landscape of NTDs in China. It presents an encompassing view of the pathogens, prevalence, the number of new infections, endemic regions, key transmission drivers, and the quantified health impacts that DW represents. For instance, the table notes significant figures such as over 80,000 local cases of dengue reported between 2005 and 2020, including 778 in 2020, with transmission chiefly facilitated by *Aedes* spp. mosquitoes and human mobility. With a 100% mortality rate, rabies reported 202 new cases in 2020. Bacterial infections like leprosy showed 200 new cases in 2020, mainly in the southern region. The table further details intervention strategies across these diseases, from mosquito breeding control and vaccination against rabies to multidrug therapy for leprosy and improved sanitation and water safety to combat schistosomiasis. These interventions align with WHO and China-specific goals, such as aiming for no global fatalities from dengue by 2030 and eliminating leprosy transmission risk and cases by the same year. For example, interventions for dengue include mosquito breeding control, prevention of local transmission, and the development of vaccines and drugs. For rabies, strategies include the vaccination of humans and dogs, and the implementation of legislation to control dog breeders. The multifaceted approach to combating schistosomiasis includes providing safe water, strengthening sanitation, and continuing snail control efforts.

### Rankings and geographic differences of the NTD burdens

**Fig. 1:**
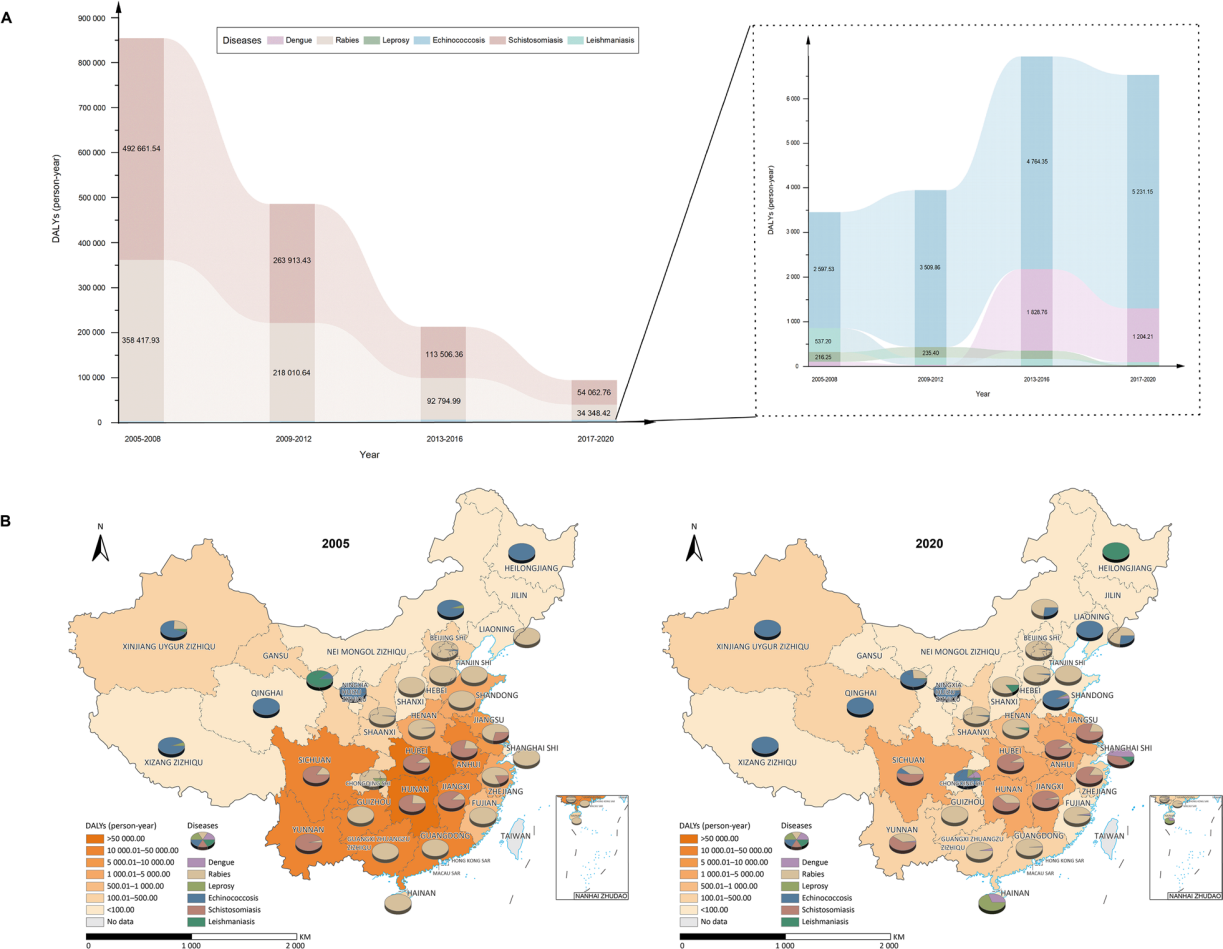
Comprehensive analysis of the burden of neglected tropical diseases (NTDs) in China from 2005 to 2020. **A** Depicts the changing trends in the overall NTDs burden within China over 16 years, presenting a declining trajectory with specific diseases highlighted at various intervals. **B** Illustrates the geographical distribution of the NTDs burden by province for the years 2005 and 2020, highlighting the variation and reduction in disease prevalence. The maps show a significant decline in the burden of NTDs in many provinces, particularly in central and southern regions of China. Map approval number: GS(2024)3052

The overall NTD burdens in China have declined significantly, as depicted in Fig. 1A. The total DALYs have decreased from 245,444.53 in 2005 to 18,984.34 in 2020, which translates to a 92.27% reduction. In 2005, the DALYs caused by schistosomiasis and rabies represent a substantial proportion of the total disease burden, accounting for 65.37% and 34.43% respectively. At the same time, those from echinococcosis, leprosy, visceral leishmaniasis, and dengue account for a smaller share. Although the disease burden of schistosomiasis and rabies is decreasing yearly, their rankings remain in the top one and two. Dengue and echinococcosis rank relatively low, but their burden is increasing continuously. The rank of dengue exceeded from the sixth to the fourth position. As shown in Fig. 1B, the downward trend in disease burden was particularly prominent in the central regions of China, including PLADs such as Hubei, Hunan, and Jiangxi, where the reductions were 94.36%, 92.54%, and 91.73% respectively. These regions mainly reported diseases of schistosomiasis, rabies, and cysticercosis.

**Fig. 2:**
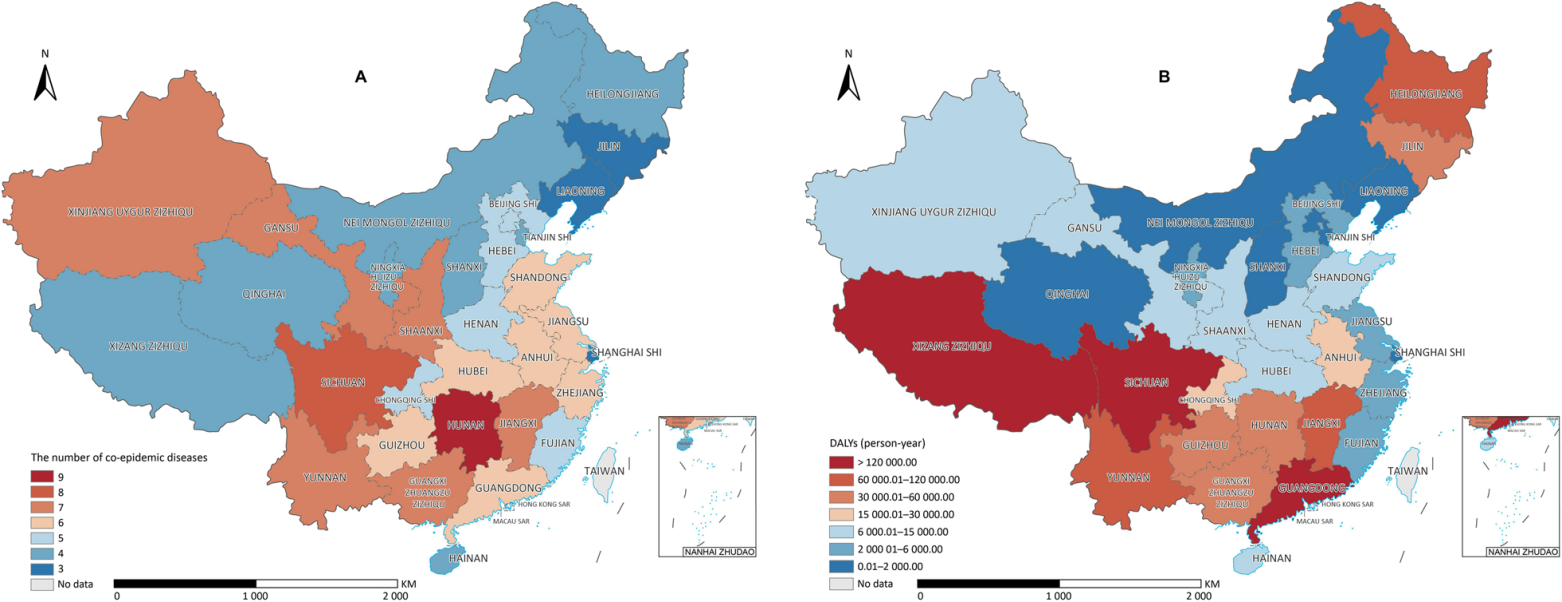
Co-endemic maps of major neglected tropical diseases (NTDs) in China by provincial-level administrative divisions in 2015. **A** The number of co-endemic NTDs. **B** The DALYs of co-endemic NTDs. In this comparison, we only included nine diseases for which data were available in our calculations, namely dengue fever, rabies, leprosy, echinococcosis, schistosomiasis, leishmaniasis, STHs, taeniasis, and clonorchiasis. Map approval number: GS(2024)3052

Given the NTDs data in 2015, including data from the third national parasitic diseases surveys, we created geographical distribution maps for the number of co-endemic diseases by PLAD and the disease burden values for the major NTDs. The PLADs with the highest diversity of NTDs (with 9 to 7 co-endemic NTDs) are in Hunan (9), Sichuan (8), Yunnan (7), Jiangxi (7) provinces and the Xinjiang Uygur Zizhiqu (Autonomous Region) (7) (Fig. 2A). Those with higher NTD burdens are placed in Sichuan and Guangdong provinces and the Xizang Zizhiqu (Fig. 2B).

**Fig. 3:**
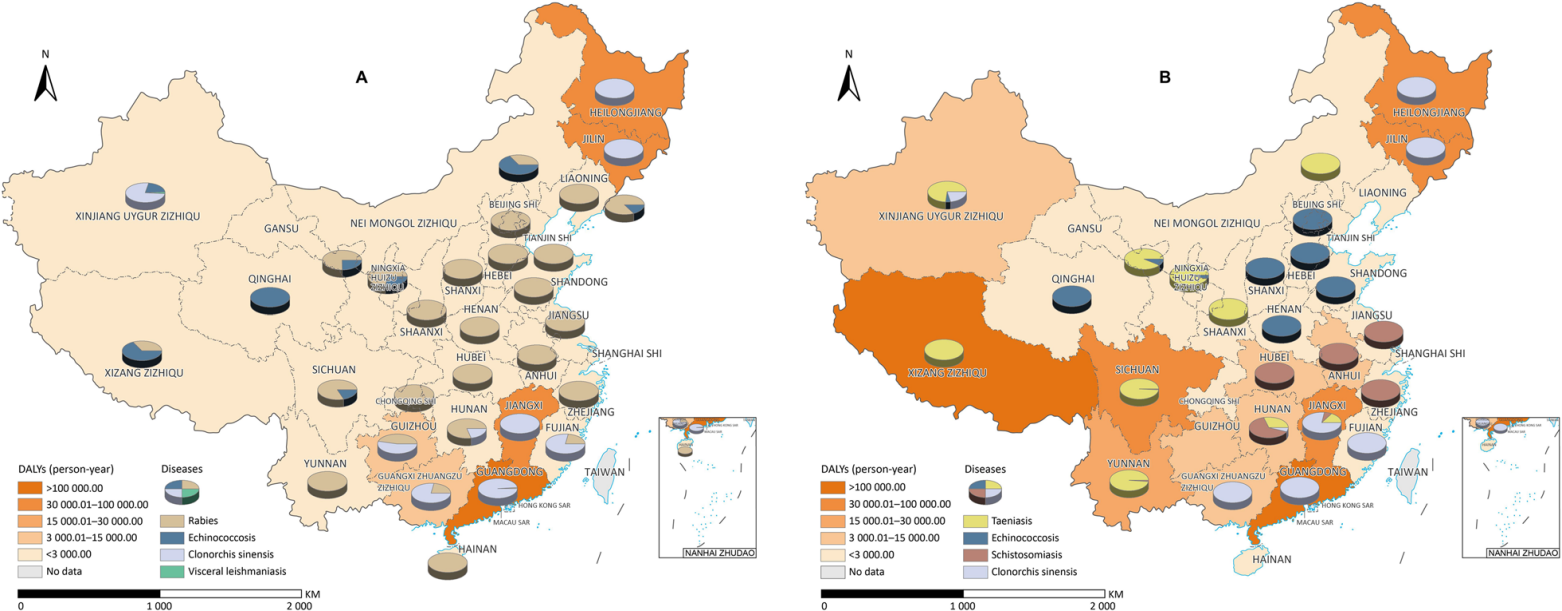
The distribution and proportion of disease burden of four dog-mediated neglected tropical diseases (NTDs) and four praziquantel-treatable NTDs. **A** Illustrates the distribution and disease burden of four dog-mediated NTDs in China. **B** Depicts the distribution and disease burden of four praziquantel-treatable NTDs, providing insights into the effectiveness of intervention strategies across different regions. Map approval number: GS(2024)3052

We know that the same host can transmit multiple diseases. For example, dogs are the primary transmission host of four NTDs: rabies, echinococcosis, leishmaniasis and clonorchiasis (Fig. 3A). In 2015, the total DALYs of the major NTDs in China numbered 840,924.60 DALYs, a number where rabies, echinococcosis, leishmaniasis and clonorchiasis accounted for 36.09% or 303,449.50 DALYs.

Praziquantel is a broad spectrum antiparasitic drug that can treat infections caused by both cestodes and trematodes (except for *Fasciola* spp. species). It is thus used against cysticercosis, echinococcosis, schistosomiasis, and the FBTs (such as clonorchiasis and paragonimiasis), which account for 57.55% of total DALYs. Xizang Zizhiqu and Guangdong province have the highest disease burden, reaching 177,707.83 and 112,109.10 DALYs, respectively. The analysis revealed that Hunan, Jiangxi, Sichuan, Yunnan provinces, and the Xinjiang Uygur Zizhiqu have a concurrent presence of 3–4 of the NTDs above (Fig. 3B).

### Spatial autocorrelation analysis

**Fig. 4:**
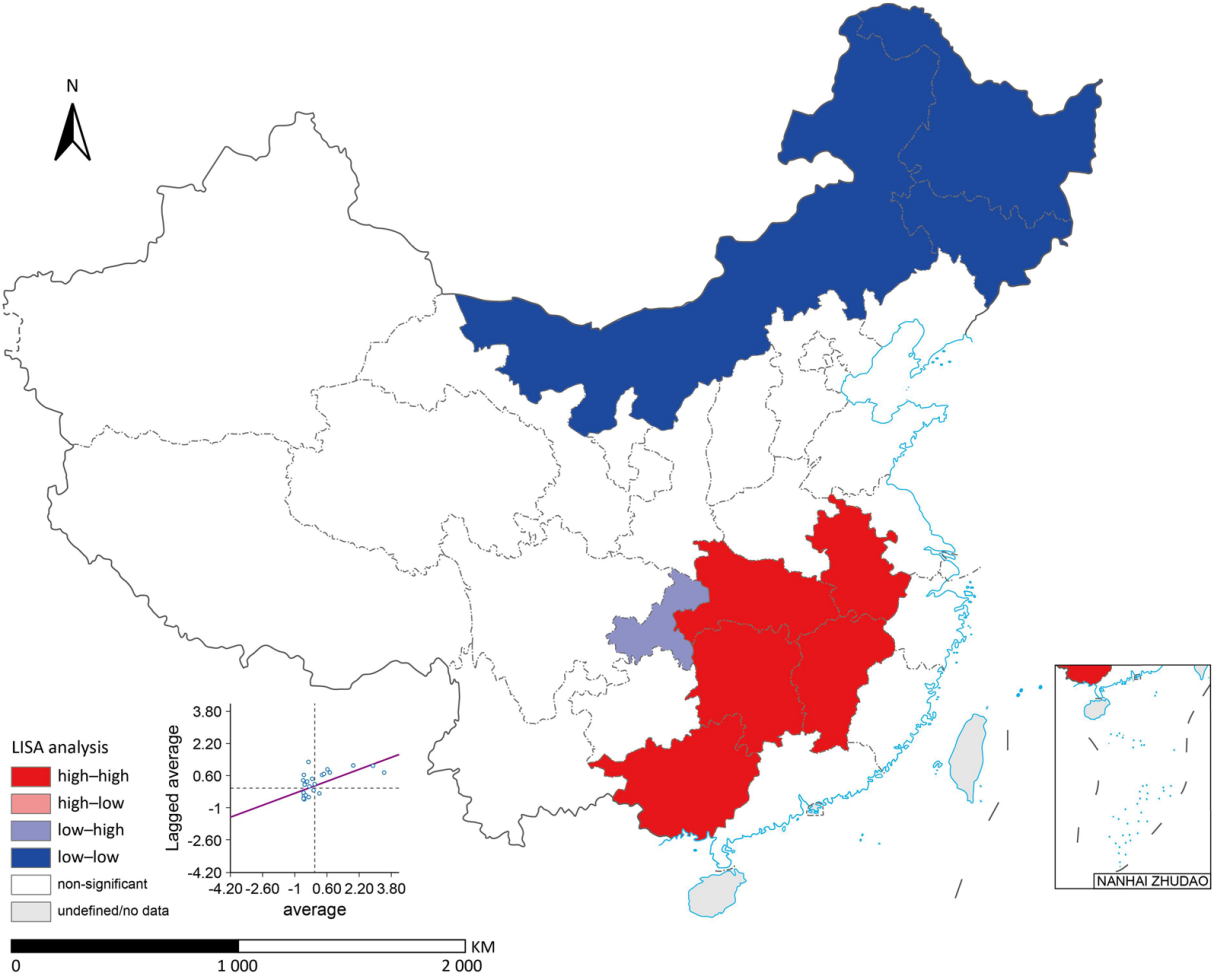
Distribution of spatiotemporal clustering of neglected tropical diseases (NTDs) burden in China from 2005 to 2020. The Global Indication of Spatial Autocorrelation coefficient (Moran's *I* = 0.371, *P* = 0.004) is presented in the scatterplots. The Local Indication of Spatial Autocorrelation outcome, based on the average NTDs burden data for the years 2005 to 2020, is depicted in China map. Red colour indicates areas with "high-high" categories and blue areas with "low-low" categories. Map approval number: GS(2024)3052

Because the regions with high and low NTD burdens did not change significantly from 2005 to 2020, we used average data for the spatial analysis. Figure 4 displays GISA and LISA clustering analysis for the average burden of NTDs from 2005 to 2020, indicating that PLADs with higher burdens of NTDs tended to cluster together. The "high–high" clusters, represented in red, include Jiangxi, Hubei, Anhui, and Hunan provinces, indicating a consistently higher burden of NTDs over the 16-year period. The "low–low" clusters, represented in blue, include PLADs in the northern regions of China, indicating a consistently lower burden. The spatial clustering pattern underscores the persistent and localized nature of the NTD burden, predominantly concentrated in central and southern China.

## Discussion

### Disease burden as an observational measure

Compared with the study carried out by Yang et al. in 2014 [18], the NTDs mapping presented here, using DALYs instead of absolute numbers of those affected, provides a more comprehensive assessment. This mapping aligns with global health goals, such as those outlined by the WHO. It would not only aid the efficient allocation of resources for NTDs control and elimination but also allow a more accurate understanding of the impact of the NTDs on society in a specific geographical region. In addition, it promotes evidence-based decision-making by considering the severity of different disabilities and the duration of time individuals are affected. Indeed, precision mapping for national NTD control programmes could assist in comparing the relative impact of different NTDs and guide resource allocation more correctly than otherwise. By prioritizing diseases with higher DALY values, limited resources can be correctly allocated and achieve better impacts. For instance, Fig. 2A demonstrates a low co-presence of 3–4 NTDs in the Xizang Zizhiqu, while Fig. 2B shows a high disease burden of 178,318.99 DALYs due to taeniasis/cysticercosis. Mapping NTDs by DALYs allows for better coordination and evaluation of progress towards these goals at the global level.

Our research found that dengue fever has continuously increased in recent years. There are several reasons why dengue cases have increased and expanded northward into China. With climate change, warmer temperatures and increased precipitation in previously cool regions have created suitable conditions for the mosquito species *Aedes aegypti* and *Ae. albopictus* that transmit the dengue virus. Globalization has facilitated the movement of infected individuals and vector mosquitoes. Rapid urbanization and infrastructure development, particularly in southern China, have created more conducive environments for mosquito breeding. It is now essential to strengthen the surveillance-response system to minimize the public health impact of this emerging/re-emerging disease. An integrated vector control approach should be implemented with relevant stakeholders, including local government, environmental agencies, and community organizations.

Echinococcosis is mainly distributed in pastoral areas with poor sanitation and economic conditions (such as the Xizang Zizhiqu, Qinghai, and Gansu provinces). It is common for Xizang nomads to feed the internal organs of slaughtered cattle and sheep to their dogs. This practice, along with weak preventive awareness, poor local sanitation conditions, and wild sources of infection (through stray dogs, wolves, and foxes), pose a severe challenge to preventing and controlling echinococcosis. It is shown that there has been an increasing trend in the number of echinococcosis cases over the years with focus in Qinghai and Sichuan provinces. It may be related to the intensive screening for echinococcosis after release of national-level control plans. Additionally, the transmission of echinococcus is influenced by various environmental factors that can be affected by natural events, including global warming. This suggests that environmental, biological, and socioeconomic factors should be considered in disease management. This is essential for the development of effective control and preventive measures to prevent the spread of echinococcosis in affected areas.

Food-borne NTDs include FBTs and taeniasis. In 2015, the disease burden of important FBTs in China accounted for 26.50% of the total NTDs in this study, suggesting that increased attention is warranted. According to the "2015 National Survey on the Status of Key Human Parasitic Diseases" [19], a total of 18 PLADs prevailed with *Clonorchis sinensis* with 3.46 million estimated infections mainly in southern and northeastern China. The disease burden of taeniasis in 2015 was estimated to be 305,320.57 DALYs, mainly in the southwestern Xizang Zizhiqu and Sichuan province. In order to promote the prevention and control of food-borne NTDs and explore control strategies under the new settings, since 2019, a total of 10 clonorchiasis intervention pilots have been established in six PLADs, namely Jilin, Heilongjiang, Hunan, Guangdong, Guizhou and Guangxi Zhuang Zizhiqu, and one pilot for taeniasis intervention in Xizang Zizhiqu [22].

**Fig. 5:**
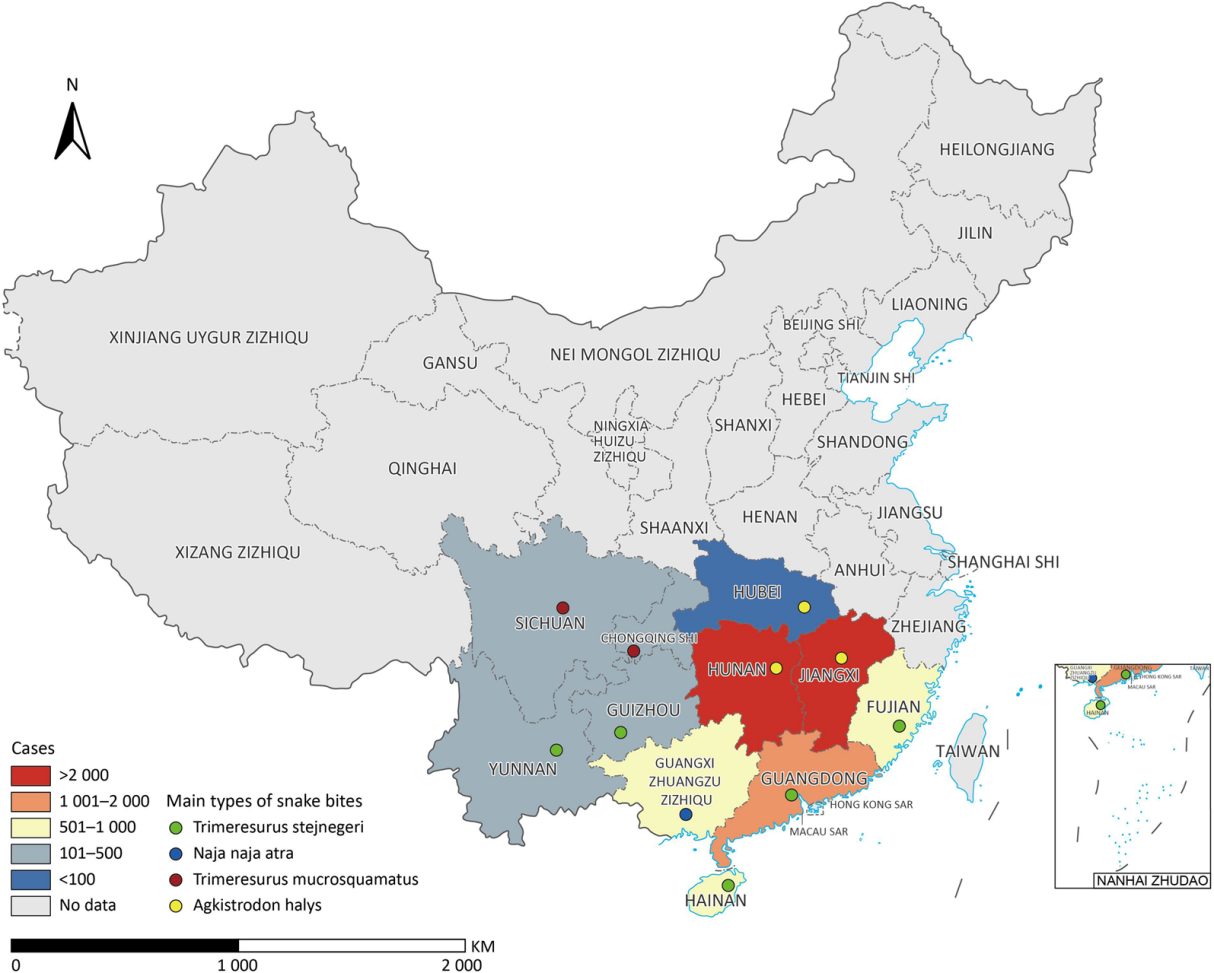
Snake bites prevalence and main types involved in surveillance provincial-level administrative divisions in China. This figure maps out the prevalence of snake bites across China's provincial-level administrative divisions. It further details the primary types of venomous snake species responsible for these bites. Map approval number: GS(2024)3052

The First National Epidemiological White Paper on Snakebite [23], published in 2023, provides an overview of the snakebite epidemiology in China (Fig. 5). The white paper presents comprehensive data on the incidence and distribution of snakebite cases in 12 PLADs. It analyses geographical variations, seasonal patterns, demographic characteristics of snakebite victims, snake species and venom profiles, healthcare access and response. Snakebite incidents in Yunnan, Guangdong, Hainan, Guizhou, and Fujian provinces are primarily attributed to *Trimeresurus stejnegeri*. Hubei, Hunan, and Jiangxi provinces, snakebites mainly involve *Agkistrodon halys*. *Protobothrops mucrosquamatus* snake is the primary culprit in Sichuan province and Chongqing Municipality. In Guangxi Zhuang Zizhiqu, *Naja atra* is the predominant species. The white paper discusses existing prevention and control measures implemented in China and highlights the importance of future key research areas. These include prioritizing advancements in the production, accessibility, and efficacy of antivenom to enhance the treatment of snakebite cases. Additionally, there is a need for ongoing monitoring snakebite incidents to comprehensively understand the evolving patterns and risk factors associated with snakebites across diverse regions. Furthermore, it emphasizes the necessity of fortifying healthcare systems to ensure prompt response and efficient access to effective treatment options.

### Harnessing the power of One Health by integrating NTD control strategies

One Health is a collaborative, multi-disciplinary approach that recognizes the interconnectedness of human, animal, and environmental health. Applying the One Health theory allows for a holistic and interdisciplinary approach to addressing zoonotic diseases [24]. It emphasizes the collaboration and coordination of experts from various fields, such as medicine, veterinary sciences, ecology, environmental sciences, and public health. By integrating knowledge and expertise from these disciplines, it becomes possible to understand the complex interactions between humans, animals, and the environment and develop cost-effective strategies for prevention and control of NTDs.

A successful One Health case in China is the control of schistosomiasis. Implementing the National Schistosomiasis Control Program in China has involved collaboration between health, agriculture, water resources, environmental protection, and health education sectors. This comprehensive approach has included snail control programs, sanitation and safe water supply improvements, health education, and regular medical treatments. These efforts with One Health approach have significantly reduced the burden of schistosomiasis in China. The disease has been eliminated as a public health problem with prevalence less than 1% by 2015 [25] and now the Chinese government has set a target to achieve the ultimate goal of complete elimination by 2030 [2].

The "Action Plan for the Prevention and Control of Echinococcosis (2010–2015)" [26] was a comprehensive initiative launched by the Chinese government to address the prevention and control of echinococcosis. Subsequently, the "National Key Parasitic Disease Prevention and Control Plan (2006–2015)" [26] and the "Healthy China Action Plan (2019–2030)" [27] were successively introduced to anti-echinococcosis. China aims to achieve the interim goal of transmission control of echinococcosis by 2025 and the goal of transmission interruption by 2030. The key components of these action plans included: (1) surveillance and monitoring of echinococcosis in both humans and animals; (2) implementation of preventive measures, such as health education and deworming programs for dogs; (3) improved diagnosis and treatment of echinococcosis in humans; and (4) coordination and collaboration among various sectors, including health, agriculture, and environmental protection, another good example reflecting One Health approach. It is believed that China will make more significant achievements in global health governance and provide other countries with Chinese solutions in the future. At the same time, considering Chinese large geographical scale and significant ecological diversity, the allocation and management of resources in the country are facing more complex challenges, and it is hoped that relevant international policies will be more inclined to support China and provide more resources.

Our research contributes valuable insights that can inform a more comprehensive and effective response to the challenges posed by NTDs, ultimately fostering shared health and sustainable development for humans, animals, and environment. In this study, we presented aggregated multiple diseases burden mapping, which allows for a comprehensive understanding of their distribution and overlaps. This information can help prioritize and allocate resources effectively and correctly by identifying common transmission pathways. Implementing intervention with One Health approach that target same host responsible for transmitting multiple diseases can be a cost-effective approach akin to a "one stone, two birds" strategy. Similarly, treating multiple NTDs with a single drug can also have a synergistic effect. This indicates that we can improve the efficiency of medical resource utilization by treating multiple diseases with a single drug. For instance, dogs serve as the primary transmission hosts for four types of NTDs**,** including rabies, echinococcosis, leishmaniasis, and clonorchiasis. Praziquantel can be used to against cysticercosis, echinococcosis, schistosomiasis, and the FBTs (such as clonorchiasis and paragonimiasis). Different diseases control groups/programmes focusing on the same target host or same drug chemotherapy should work together to effectively interrupt the transmission of these NTDs. Health education and targeted awareness campaigns should be conducted to educate key populations about the risks and harms associated with NTDs transmitted by same host and treated by same drug.

### Study limitations

This study has several limitations. Firstly, the reliance on reported cases and deaths from national databases may result in underreporting or misreporting, leading to potential inaccuracies in the disease burden estimates. Secondly, the use of disability weights from global sources may not fully capture the local context and specific impacts of NTDs in China. Furthermore, the burden of mental health/disability [28] is not considered in this study, which may lead to an underestimation of the actual disease burden. Additionally, the data on NTDs were obtained from various sources, and the quality and completeness of the data may vary, potentially affecting the accuracy of our estimates.

## Conclusions

This study employs DALYs as the core metric to deeply analyse the disease burden of NTDs. Compared to traditional research methodologies based on morbidity rates, the use of DALYs offers a more comprehensive assessment perspective, guiding the effective control and eradication of NTDs. We learned that China's significant progress in the fight against NTDs is multifaceted. Firstly, through national health reforms and public health initiatives, the disease burden of major NTDs in China has been significantly reduced. For example, schistosomiasis has implemented a comprehensive disease surveillance and control strategy, and the incidence and transmission of the disease has been greatly reduced. Additionally, substantial investments by the Chinese government in infrastructure and public health services have positively impacted disease control efforts. Secondly, the adoption of the One Health concept to guide disease prevention and control is an innovative approach. This method not only improves the utilization efficiency of medical resources, but also promotes the sustainable development of public health sector. Finally, China's success story provides valuable experience and models for other countries to learn from. With strong government policy support and multi-sectoral cooperation, China has demonstrated the feasibility of effectively controlling NTDs, providing important insights for global health governance.

## Supplementary Information

**Additional file 1: Table S1.** DALYs (person-years) of neglected tropical diseases in China, 2005-–2020.

## Data Availability

Relevant data are available from the corresponding author on request.

## Abbreviations

NTDs: Neglected tropical diseases
CDC: Center for Disease Control and Prevention
DALYs: Disability-adjusted life years
YLLs: Years of life lost
YLDs: Years lived with disability
WHO: World Health Organization
FBTs: Food-borne trematodes
CNKI: China National Knowledge Infrastructure
PLADs: Provincial-level administrative divisions
STHs: Soil-transmitted helminths
DW: Disability weights
GHDx: Global Health Data Exchange
GISA: Global Indication of Spatial Autocorrelation
LISA: Local Indication of Spatial Autocorrelation
