# Supplementary material for: Spatial-temporal distribution of neglected tropical diseases burdens in China from 2005 to 2020
Source: Infect Dis Poverty. 2024 Sep 4;13:64. doi: 10.1186/s40249-024-01235-y (PMC11373236; doi:10.1186/s40249-024-01235-y)
Supplement: Supplementary file 1 — Additional file 1: Table S1. DALYs (person-years) of neglected tropical diseases in China, 2005-–2020. [file 40249_2024_1235_MOESM1_ESM.docx]

**Supplementary Table:**

**Table S1.** DALYs (person-years) of neglected tropical diseases in China, 2005-2020

| Year | dengue | rabies | leprosy | echinococcosis | schistosomiasis | leishmaniasis |
| --- | --- | --- | --- | --- | --- | --- |
| 2005 | 51.69 | 84503.15 | 52.44 | 221.37 | 160452.38 | 163.50 |
| 2006 | 32.44 | 104820.89 | 62.74 | 388.67 | 139465.66 | 82.86 |
| 2007 | 16.75 | 99537.11 | 45.21 | 1071.52 | 106377.52 | 265.34 |
| 2008 | 6.28 | 69556.78 | 55.86 | 915.97 | 86365.98 | 25.50 |
| 2009 | 9.48 | 62100.84 | 13.73 | 808.15 | 77432.84 | 25.96 |
| 2010 | 6.93 | 62303.23 | 74.18 | 794.19 | 70107.46 | 60.74 |
| 2011 | 3.73 | 55138.86 | 13.73 | 878.52 | 62579.51 | 14.94 |
| 2012 | 17.86 | 38467.71 | 133.76 | 1029.00 | 53793.62 | 63.45 |
| 2013 | 144.87 | 33058.48 | 110.34 | 1187.31 | 42958.06 | 40.39 |
| 2014 | 1500.33 | 22594.09 | 13.61 | 1015.63 | 29917.89 | 52.17 |
| 2015 | 119.86 | 20623.47 | 47.26 | 1056.08 | 22532.00 | 25.86 |
| 2016 | 63.69 | 16518.95 | 11.08 | 1505.33 | 18098.42 | 54.40 |
| 2017 | 220.77 | 12637.77 | 11.75 | 1656.47 | 14571.14 | 9.28 |
| 2018 | 208.41 | 10421.67 | 8.79 | 1306.73 | 13108.04 | 8.16 |
| 2019 | 750.87 | 6444.98 | 9.09 | 1247.75 | 13336.10 | 7.70 |
| 2020 | 24.17 | 4843.98 | 7.80 | 1020.18 | 13047.47 | 40.73 |
| Average | 198.63 | 43973.25 | 41.96 | 1006.43 | 57759.01 | 58.81 |
| Total | 3178.13 | 703571.96 | 671.37 | 16102.87 | 924144.09 | 940.98 |

Notes: value in each cell is presented as DALYs.
